# Adaptation of the Taiwan Version of the Supportive and Palliative Care Indicators Tool (SPICT-TW) and Its Association with Six-Month Mortality: A Multi-Center Validation Study in Older People

**DOI:** 10.3390/healthcare12212185

**Published:** 2024-11-01

**Authors:** Jung-Yu Liao, Hsiao-Ting Chang, Jen-Kuei Peng, Scott A. Murray, Chien-Yi Wu, Hsien-Cheng Chang, Chia-Ming Li, Shao-Yi Cheng, Wei-Zhe Tseng, Chao Agnes Hsiung, Hung-Yi Chiou, Sang-Ju Yu, Kirsty Boyd, Ping-Jen Chen

**Affiliations:** 1 Department of Health Promotion and Health Education, National Taiwan Normal University, Taipei 106, Taiwan; jyliao@ntnu.edu.tw; 2 Department of Family Medicine, Taipei Veterans General Hospital, Taipei 112, Taiwan; htchang2@vghtpe.gov.tw; 3 Department of Family Medicine, National Yang Ming Chiao Tung University, Taipei 30010, Taiwan; 4 Department of Family Medicine, College of Medicine and Hospital, National Taiwan University, Taipei 10617, Taiwan; jkpeng@ntu.edu.tw (J.-K.P.); sharlenecheng@ntuh.gov.tw (S.-Y.C.); 5 Usher Institute, University of Edinburgh, Edinburgh EH8 9YL, UK; scott.murray@ed.ac.uk (S.A.M.); kirsty.boyd@ed.ac.uk (K.B.); 6 Department of Family Medicine, Kaohsiung Medical University Hospital, Kaohsiung Medical University, Kaohsiung 807, Taiwan; chienyi@kmu.edu.tw (C.-Y.W.); r100263@kmu.edu.tw (W.-Z.T.); 7 Keelung City Health Bureau, Keelung 201, Taiwan; chief@mail.klcg.gov.tw; 8 Family Medicine Department, National Taiwan University Hospital Beihu Branch, Taipei 10617, Taiwan; b14002@bh.ntuh.gov.tw; 9 Institute of Population Health Sciences, National Health Research Institutes, Miaoli 11503, Taiwan; hsiung@nhri.edu.tw (C.A.H.); hychiou@nhri.edu.tw (H.-Y.C.); 10 Taiwan Society of Home Health Care, Taipei 100, Taiwan; bebe.yu@gmail.com; 11 Home Clinic Dulan, Taitung 959, Taiwan; 12 Marie Curie Palliative Care Research Department, Division of Psychiatry, University College London, London W1T 7NF, UK; 13 National Center for Geriatrics and Welfare Research, National Health Research Institutes, Miaoli 11503, Taiwan; 14 School of Medicine, College of Medicine, National Sun Yat-sen University, Kaohsiung 804, Taiwan

**Keywords:** home-based medical care (HBMC), older people, palliative care, Taiwan version of the Supportive and Palliative Care Indicators Tool (SPICT-TW), validation study

## Abstract

**Background:** The Supportive and Palliative Care Indicators Tool (SPICT) was developed for identifying, in a timely manner, patients who may benefit from supportive and palliative care for better treatment review, care-plan discussion, and end-of-life care. Although the SPICT has been validated in different languages and for patients living in different settings, it has not been validated for patients receiving home-based medical care (HBMC), or in the context of using traditional Chinese characters. **Objectives:** The present study aimed to validate the Taiwanese version of the SPICT (SPICT-TW) and to measure its ability to predict six-month mortality in patients who received HBMC in Taiwan. **Methods:** Seven HBMC agents (five clinics and two hospitals) participated in this validation study. We recruited 129 patients aged ≥ 50 years who had been consistently receiving HBMC for >two months. **Results:** The results revealed that the SPICT-TW demonstrated similar reliability and validity compared to other language versions of the SPICT. It may be an appropriate tool for healthcare professionals to detect, in a timely manner, the needs for palliative care in older people who receive home healthcare. Furthermore, we found that a combination of four general indicators and one clinical indicator in the SPCIT-TW has the best prediction ability at predicting six-month mortality in these HBMC recipients. This multi-center study validated the SPICT-TW among HBMC recipients in Taiwan. **Conclusions:** The SPICT-TW demonstrated high reliability and validity through the Kuder–Richardson 20, an intraclass correlation coefficient, Cohen’s kappa, and receiver operating characteristic analysis, supporting its potential as a practical tool for identifying older adults at risk of dying within six months who have not yet received palliative care but may benefit from it.

## 1. Introduction

Identifying patients who need palliative care and providing timely palliative care to them has shown benefits to these patients and their families. The Supportive and Palliative Care Indicators Tool (SPICT) was developed by Boyd et al. in Scotland in 2010 for early identification of patients who may be benefit from supportive and palliative care for better treatment review, care-plan discussion, and end-of-life care. The SPICT comprises three parts, including measures to find out indicators for poor or deteriorating health, to discover clinical indicators of life-limiting conditions, and to review current care and care planning [1]. The SPICT has been translated to different languages [2,3,4,5,6] and adapted for patients in different settings including acute wards in hospitals [7,8,9,10,11], primary care [12,13,14], and care homes [15].

Many patients with chronic conditions and limited functions are cared for at home. These patients are often living with multiple chronic conditions, having complex needs and having higher risk of deterioration as well [16,17,18]. Some of these patients receive home-based medical care (HBMC), including physician visits, skilled nursing care, and living care provided by medical care teams from hospitals or healthcare agencies in the community [19,20]. In Taiwan, HBMC services are reimbursed by the Integrated HBMC Program of the National Health Insurance [21]. The program provides different levels of HBMC services for patients with different levels of needs. However, many of these patients have not yet been evaluated for palliative-care eligibility, despite having multiple comorbidities, frailty, and a high risk of deterioration [22]. Therefore, timely identification of their palliative-care needs and a review the treatments, medications, and care plans for these patients is warranted.

Although the SPICT has been validated in different languages and for patients living in different settings, it has not been validated for patients receiving HBMC or in the context of using traditional Chinese characters. Therefore, we aimed to validate the Taiwanese version of the Supportive and Palliative Care Indicators Tool (SPICT-TW) and to measure its ability to predict 6-month mortality in patients receiving HBMC in Taiwan.

## 2. Materials and Methods

### 2.1. Study Design and Procedure

This study is a part of the Home-based Longitudinal Investigation of the Multidisciplinary Team Integrated Care (HOLISTIC) [23]. It comprises two steps, including the adaptation of the SPICT-TW and the examination of validity and reliability of the SPICT-TW among patients in the Integrated HBMC Program, involving HBMC recipients and HBMC-plus recipients. The study protocol was approved by the Research Ethics Committee of National Health Research Institutes in Taiwan (EC1080203, EC1080203-R1).

### 2.2. Adaptation of the SPICT-TW

Based on the recommendations of the World Health Organization (WHO) [24], we conducted the forward and backward translation of the original SPICT, including the 7 general health indicators, a total of 23 clinical indicators for ten specific life-limiting illnesses, and 5 items for guidelines for a palliative-care approach to the patients. Our expert panel comprised specialists in palliative medicine, geriatrics and gerontology, nursing, home healthcare, long-term care, social welfare, and public health. Following that, we piloted it among patients in the Integrated HBMC program via face-to-face interviews for the feasibility testing. Before each interview, our trained interviewers briefly explained the study protocol and described end-of-life scenarios to eligible patients and their caregivers. We conducted interviews in comfortable, safe spaces. Caregivers were allowed to accompany and support patients during interviews if needed. After the feasibility testing, the expert panel compared the back-translated English version with the original version and made suggestions based on the results drawn from feasibility testing regarding modifications to the final version.

### 2.3. The Validity and Reliability of the SPICT-TW

We explored the association between SPICT-TW scores and six-month mortality after the date of study participation and evaluated the criterion-related validity, as well as intra-rater and inter-rater reliability (refer to Statistical Analysis section for details) [25]. Therefore, HBMC and HBMC-plus recipients in this study were evaluated with the SPICT-TW twice by healthcare professionals, such as physicians, nurses, or social workers providing HBMC services. The two assessments for each patient needed to be conducted by the same healthcare professional and the interval between them was less than one month. In order to examine the inter-rater reliability of the SPICT-TW, some patients were assessed by both the physician and another healthcare professional (e.g., nurse or social workers) simultaneously at the two timepoints.

### 2.4. Other Measurements

Besides demographic characteristics, we additionally collected data about previous hospital utilization, as well as data from the five-item WHO Well-Being Index (WHO-5) and the Clinical Frailty Scale (CFS) at baseline. The WHO-5 assessed patients using five statements, which respondents rated according to the scale from “at no time” (0) to “all of the time” (5) in relation to the past two weeks [26]. The total raw score ranged from 0 to 25. A score of 13 was used as the cut-point for which the scores lower or higher than the cut-point represented good or bad well-being, respectively.

The CFS, a judgement-based frailty tool, generates a frailty score ranging from 1 (very fit) to 9 (terminally ill) by specific domains including comorbidity, function, and cognition [27]. Participants were assessed with the CFS and categorized into four groups, including “without frailty/ mild frailty” (score ≤ 5), “moderate frailty” (score of 6), “severe frailty” (score of 7), and “very severe to terminal ill” (score of 8–9).

### 2.5. Participants

A total of 7 out of 18 agents in the HOLISTIC study (5 clinics and 2 hospitals) participated in this validation study. We recruited patients in the aforementioned seven agents who met the following inclusion criteria: (1) aged ≥ 50 years, (2) had been consistently enrolled in the Integrated HBMC Program for more than two months, and (3) had cognitive impairment but were supported by cognitively competent caregivers to facilitate communication. Patients who were unwilling to provide informed consent were excluded from the study. In Taiwan, HBMC is mainly provided by physician visits for patients who have limited activities of daily living (Barthel index score less than 60) or who have difficulty visiting healthcare agents due to disease conditions. HBMC-Plus comprises physician, nurse, respiratory therapist, and pharmacist visits to patients whose disability is more severe than those in HBMC, having definite medical or nursing care needs such as “change tracheostomy set”, “urinal indwelling catheterization”, “insertion of nasogastric tube”, “bladder irrigation”, “wound treatment”, “intravenous drip”, and “colostomy irrigation” that are assessed by both physicians and nurses as chronic conditions requiring long-term nursing care or having continual post-discharge healthcare needs [19].

### 2.6. Statistical Analysis

Data were imported into Excel and managed and analyzed in SAS (version 9.4; SAS Institute, Inc., Cary, NC, USA). Statistical significance was set at a *p*-value < 0.05 [28]. Missing data were not imputed. We used the Kuder–Richardson 20 (KR-20), an intraclass correlation coefficient (ICC), and Cohen’s kappa as indicators for internal consistency reliability, intra-rater reliability, and inter-rater reliability, respectively. The value of the KR 20 was 0.7 or higher, indicating an acceptable value of reliability. The value of ICC ≥ 0.7 was considered acceptable, and the value ≥ 0.90 was considered excellent [25]. The value of kappa ≥ 0.6 was considered acceptable, and the value ≥ 0.80 was considered excellent [29].

On the other hand, we conducted a receiver operating characteristic (ROC) analysis for the predictive validity. The sensitivities and specificities at different numbers of indicators were individually presented. Both the area under the ROC curve (AUC) and the Youden Index were used for determining the distinguishing ability of the scale and considering the optimal number of indicators for the cut-off point, whereas a χ^2^ test was used for the power of discrimination. We followed the suggestions of Hosmer and Lemeshow, in which 0.7 ≤ AUC < 0.8 indicates the acceptable discrimination and AUC ≥ 0.8 indicates excellent discrimination [30].

To examine the predictive power of the optimal number of general health and clinical indicators in the SPICT-TW on the follow-up six-month mortality, a multivariate logistic regression model was used after controlling for the age, comorbidity, HBMC types, past-30 days hospitalization, WHO-5 Well-Being Index, and CFS. Our findings and the suggested cut-off points were examined to compare the different predictive powers in the SPICT-TW on follow-up six-month mortality [7].

## 3. Results

### 3.1. Background Information of Patients and Evaluators

In total, 129 patients were assessed by 35 healthcare professionals (evaluators) with the SPICT-TW. We excluded one patient because his two assessments were conducted by different persons. The characteristics of patients are shown in Table 1. The average age was 82.4 years old (SD = 12), and two-thirds of them had five or more comorbidities. Compared with the HBMC-Plus recipients, HBMC recipients had better well-being (*p* < 0.001) and less frailty (*p* < 0.001). Furthermore, the main common diseases were different between the HBMC patients and HBMC-Plus patients. Compared with the HBMC patients, the HBMC-Plus patients had higher percentage rates of pressure injury (46% vs. 13%) and Parkinson’s disease (26% vs. 12%). Hypertension was the most common disease in both HBMC and HBMC-Plus patients.

These 35 evaluators comprise 11 physicians (31.4%), 19 nurses (54.3%), and 5 other healthcare professionals (e.g., social workers or case managers) (14.3%) (Table 2). A total of 60% of them worked at hospitals, and their median of work experience was 15 years (IQR: 4.6–24.5).

### 3.2. The Reliability and Validity of the SPICT-TW

Table 3 shows the results regarding internal consistency reliability and intra-rater reliability. The overall KR-20 of the SPICT-TW scale ranging from 0.77 to 0.86 as performed by evaluators in different disciplines indicated an acceptable value of internal consistency reliability. The value of ICC was 0.92 (95% CI 0.89–0.95), which indicated consistency between the two assessments. Moreover, the inter-rater reliability was examined, and values of Cohen’s kappa were higher than 0.6 in most of the indicators (86.7%).

The sensitivity, specificity, and AUC of the number of general health indicators and clinical indicators are shown in Table 4. The AUCs of general health indicators and clinical indicators were 0.73 and 0.61 respectively between patients with and without six-month mortality, indicating an acceptable validity. It was significant among general health indicators and a cut-off value of 4 for the general health indicators had the highest Youden index value. However, we found AUCs of clinical indicators is nonsignificant.

**Figure 1:**
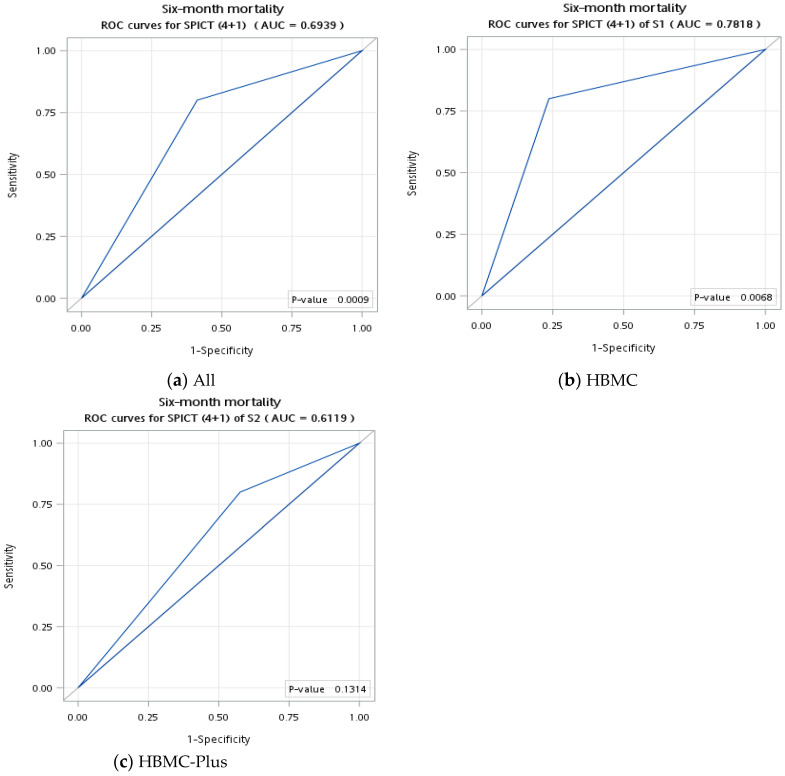
Receiver-operating characteristic curve of SPICT (4 + 1) for six-month mortality among (**a**) all patients, (**b**) HBMC patients, and (**c**) HBMC-Plus patients. HBMC = Home-based Medical Care, SPICT = Supportive and Palliative Care Indicators Tool. The cut-off score of AUC was 0.7. Statistical significance was set at a *p*-value < 0.05.

Using the optimal values for general health indicators and clinical indicators, we conducted the subgroup analysis of the ROC curve among HBMC patients and HBMC-Plus patients. The value of AUC among HBMC recipients was better than HBMC-Plus patients (0.78 vs. 0.61) (Figure 1).

### 3.3. Association Between the SPICT-TW and Six-Month Mortality

The results of univariate and multivariate logistic regressions for predicting six-month mortality are shown in Table 5. We examined two models with different definitions of SPICT-positive patients. In model I, the SPICT-positive patients were identified with the aforementioned findings (a cut-off value of 4 for general health indicators and a cut-off value of 1 for clinical indicators) in the Taiwanese context. In model II, the SPICT-positive patients were identified with a cut-off value of 2 for general health indicators and a cut-off value of 1 for clinical indicators based on the original identification approach of the SPICT.

Based on Table 5, patients who were identified with a cut-off value of 4 for general health indicators and a cut-off value of 1 for clinical indicators in the SPICT-TW were at significantly higher risk of follow-up 6-month mortality (odds ratio (OR) = 8.30, *p* = 0.034). The other variables were not significant. It indicated that the SPICT-TW had a predictive power of acceptableness regarding follow-up six-month mortality, and its optimal cut-off number of indicators is a combination of four general health indicators and one clinical indicator.

## 4. Discussion

This is the first validation study of the SPICT in the Taiwanese context using traditional Chinese characters and taking place in HBMC settings. The SPICT-TW demonstrated similar reliability and validity compared to other language versions of the SPICT. It may be an appropriate tool for healthcare professionals to, in a timely manner, detect the need for palliative care in older people who receive home healthcare. Furthermore, we found that a combination of four general indicators and a clinical indicator in the SPCIT-TW has the best prediction ability at predicting 6-month mortality in these HBMC recipients.

Based on the findings of reliability, the internal consistency reliability of the SPICT-TW was assessed by the KR-20, and it was found that the acceptable results were consistent with a previous study in Europe [2]. We additionally found that it was not affected by different disciplines of healthcare professionals (e.g., physicians, nurses, and social workers) at any time. Both the intra-rater reliability and inter-rater reliability were examined and found to be at good levels, indicating that assessment via the SPICT-TW was consistent and reliable.

Studies have demonstrated that the SPICT has good value regarding identifying older patients at high risk of health degradation and mortality, although the tool was not developed for prognostic purposes [8,10,31]. In this study, compared to previous hospitalization, WHO-5 data, CFS data, and numbers of comorbidities, the SPICT-TW positive was the only significant scale associated with six-month mortality in the multivariable analysis. However, we found that a cut-off point of four general health indicators plus one clinical indicator in the SPICT-TW had better association with six-month mortality in the Taiwanese cohort. De Bock et al. conducted a retrospective cohort study in Belgium and found that, among inpatients in an acute geriatric ward at a university hospital, a cut-off value of two general indicators and a clinical indicator in the SPICT successfully predicted one-year mortality with a sensitivity of 0.841, and AUCs of the general indicators (0.76) and the clinical indicators of the SPICT (0.75) did not significantly differ [8]. In contrast, among the HBMC patients in our study, the conditions are chronic and may be less unstable, so we need more general indicators of the SPICT-TW to increase the value of survival prediction. Furthermore, regarding the association with six-month mortality, the AUCs of the general indicators (0.73) in our study were higher than those of clinical indicators (0.61).

HBMC-Plus patients were significantly frail and had poor well-being compared to HBMC patients, and the SPICT-TW 4 + 1 was found to have better prediction of 6-month mortality in HBMC than in HBMC-Plus patients. There might have complex mechanisms for this association, including the type of comorbidity, the severity of comorbidity, the functional status of these patients, the frequency of condition fluctuations, etc. It may also reveal that HBMC and HBMC-Plus include different patient groups, indicating that while considering the follow-up six-month mortality, we should consider different combinations of general indicators and clinical indicators. Moreover, for early identification of palliative-care needs, further evaluation of HBMC and HBMC-Plus patients who live with different comorbidities or multimorbidities, should be considered via comprehensive assessments, along with their psychosocial and spiritual well-being.

The strength of our study resides in its enrollment from a national cohort, employing stratified sampling across Taiwan, which enhances the generalizability of its findings. The involvement of diverse healthcare professionals, including physicians, nurses, and social workers, ensures that the SPICT-TW’s reliability and applicability were tested across different disciplines. Additionally, the study in HBMC settings provides practical insights, particularly for elderly patients with multiple chronic conditions. However, there are still several limitations. First, the study focused on six-month mortality, which may not capture long-term outcomes and the full impact of palliative-care interventions initiated based on SPICT-TW assessments. Second, the severity of diseases or clinical conditions related to mortality were not evaluated, which might have underestimated the patients’ needs for palliative care. Third, although efforts were made to ensure consistency, the subjective nature of some assessments could introduce bias, particularly in the inter-rater reliability evaluations. Fourth, while the study assessed mental well-being and frailty using the WHO-5 Well-Being Index and CFS scale, it did not incorporate other important aspects of palliative care, such as patients’ spiritual well-being. Future research could benefit from incorporating additional tools to provide a more comprehensive evaluation. Lastly, conducting validation research in such busy clinical care settings was a challenge, limiting our ability to engage more physicians, nurses, and other members of the Integrated HBMC Program teams in evaluating the SPICT. Although the sample size of 129 participants was sufficient for initial validation, it may not be large enough to conduct analyses across subgroups such as gender, comorbidities, or age groups. Future studies can assess the sensitivity and specificity of the SPICT in larger, more diverse populations to enhance its accuracy and broader applicability.

## 5. Conclusions

This multi-center study validated the SPICT-TW among HBMC recipients in Taiwan. The SPICT-TW demonstrated high reliability and validity and may be a practical tool that can be used for identifying older people at risk of dying within six months who would benefit from palliative care. Future research should explore the effectiveness of the SPICT in initiating timely palliative care and its practical value for the patients’ quality of life, symptom improvement, and caregiver burden.

## Figures and Tables

**Table 1:** Background information of study population.

	Total, *n* = 129	Types in the Integrated HBMC Program		
		HBMC, *n* = 60	HBMC-Plus, *n* = 69	*p*-Value
Age, mean (SD)	82.4 (12.0)	81.7 (11.9)	83.0 (12.2)	0.516
Sex (female), *n* (%)	69 (53.5)	33 (55.0)	36 (52.2)	0.748
Comorbidity, *n* (%)				0.259
<5	43 (33.3)	22 (36.7)	21 (30.4)	
5–6	49 (38.0)	25 (41.7)	24 (34.8)	
≥7	37 (28.7)	13 (21.7)	24 (34.8)	
Hospitalization in past 30 days, *n* (%)	13 (10.0)	3 (5.0)	10 (14.5)	0.086
WHO-5, *n* (%)				<0.001
Good	64 (49.6)	40 (66.7)	24 (34.8)	
Poor	65 (50.4)	20 (33.3)	45 (65.2)	
Clinical Frailty Scale, *n* (%)				<0.001
≤5	22 (17.1)	17 (28.3)	5 (7.3)	
6	34 (26.4)	26 (43.3)	8 (11.6)	
7	48 (37.2)	13 (21.7)	35 (50.7)	
8–9	25 (19.4)	4 (6.7)	21 (30.4)	
Six-month mortality, *n* (%)	15 (12.3)	5 (8.3)	10 (14.5)	0.276

HBMC = Home-based medical care, WHO-5 = World Health Organization-5 Well-Being Index. Statistical significance was set at a *p*-value < 0.05.

**Table 2:** Background information of evaluators.

	Total (*n* = 35)
Age, mean (SD) ^a^	40.6 (10.0)
Years of work experience, median (IQR) ^b^	15 (4.6–24.5)
Professional of evaluator, *n* (%)	
Physician	11 (31.4)
Nurse	19 (54.3)
Others	5 (14.3)
Type of affiliation, *n* (%)	
Hospital	21 (60.0)
Clinic	14 (40.0)

IQR = interquartile range. ^a^ missing (*n* = 4), ^b^ missing (*n* = 3).

**Table 3:** Internal consistency reliability and intra-rater reliability of SPICT-TW.

	SPICT-TW		
	All Indicators	General Health Indicators	Clinical Indicators
KR-20			
All (*n* = 129)	0.84	0.67	0.80
Physician (*n* = 53)	0.83	0.61	0.77
Nurse (*n* = 55)	0.86	0.66	0.82
Others (*n* = 21)	0.77	0.71	0.80
ICC (95% CI)	0.92 (0.89–0.95)	0.91 (0.87–0.93)	0.87 (0.82–0.91)

ICC = intraclass correlation coefficient, KR-20 = Kuder–Richardson 20, SPICT-TW = Taiwanese version of the Supportive and Palliative Care Indicators Tool. The cut-off score of KR-20 and ICC was 0.7.

**Table 4:** Receiver Operating Characteristic Analysis in the general and clinical indicators of the Supportive and Palliative Care Indicators Tool for six-month mortality.

	Number of Indicators	Sensitivity	Specificity	Youden Index	AUC (95% CI)
**General indicators**	1	0.93	0.07	0.003	0.73 (0.58–0.88)
	2	0.93	0.18	0.108	*p* = 0.002
	3	0.87	0.36	0.227	
	4	0.80	0.56	0.361	
	5	0.53	0.83	0.358	
	6	0.20	0.97	0.165	
	7	0.07	0.99	0.058	
**Clinical indicators**	1	0.87	0.25	0.113	0.61 (0.46–0.76)
	2	0.67	0.49	0.158	*p* = 0.154
	3	0.20	0.90	0.095	
	4	0.13	0.98	0.115	
	5	0.13	0.99	0.124	
	6	0.07	1.00	0.067	

AUC = area under the curve, CI = confidence interval. Statistical significance was set at a *p*-value < 0.05. The cut-off score of AUC was 0.7. Youden index was selected with the highest score.

**Table 5:** Multivariable logistic regression model for six-month mortality.

Variable	Univariate Model			Full Model I			Full Model II		
	OR	95% CI	*p*	OR	95% CI	*p*	OR	95% CI	*p*
SPICT (4 + 1)									
Negative	[Ref]			[Ref]			N/A		
Positive	5.70	(1.53–21.3)	0.010	8.30	(1.17–58.82)	0.034			
SPICT (2 + 1)									
Negative	[Ref]			N/A			[Ref]		
Positive	3.12	(0.67–14.55)	0.147				6.24	(0.57–68.29)	0.134
Age									
<75	[Ref]			[Ref]			[Ref]		
75–84	1.92	(0.19–19.6)	0.583	1.16	(0.08–16.01)	0.914	1.34	(0.11–17.18)	0.822
≥85	4.60	(0.56–37.7)	0.155	4.46	(0.42–47.28)	0.215	5.11	(0.50–52.31)	0.169
Sex									
Male	[Ref]			[Ref]			[Ref]		
Female	0.99	(0.34–2.92)	0.990	0.34	(0.08–1.54)	0.162	0.31	(0.07–1.44)	0.134
Comorbidity									
<5	[Ref]			[Ref]			[Ref]		
5–6	0.55	(0.14–2.09)	0.378	0.38	(0.07–2.08)	0.263	0.33	(0.06–1.70)	0.184
≥7	0.96	(0.27–3.46)	0.955	1.19	(0.21–6.74)	0.848	0.95	(0.18–5.17)	0.954
HBMC type									
HBMC	[Ref]			[Ref]			[Ref]		
HBMC-Plus	1.86	(0.60–5.80)	0.282	0.49	(0.09–2.57)	0.395	0.56	(0.11–2.87)	0.491
Hospitalization in past 30 days									
No	[Ref]			[Ref]			[Ref]		
Yes	2.60	(0.63–10.78)	0.188	2.14	(0.31–14.88)	0.441	2.75	(0.37–20.49)	0.323
WHO-5 Well-Being Index									
Good	[Ref]			[Ref]			[Ref]		
Poor	4.60	(1.23–17.2)	0.023	3.09	(0.50–19.13)	0.226	3.24	(0.56–18.62)	0.188
Clinical Frailty Scale									
1–5	[Ref]			[Ref]			[Ref]		
6	2.03	(0.20–20.89)	0.551	0.63	(0.04–11.07)	0.754	0.82	(0.05–13.44)	0.888
7	0.91	(0.08–10.64)	0.942	0.09	(0.01–2.85)	0.170	0.12	(0.01–3.74)	0.229
8–9	11.8	(1.35–103.04)	0.026	2.14	(0.09–49.52)	0.636	2.70	(0.12–60.56)	0.531

OR = odds ratio, Ref = reference group, HBMC = home-based medical care, SPICT = Supportive and Palliative Care Indicators Tool. Statistical significance was set at a *p*-value < 0.05.

## Data Availability

The datasets presented in this article are not readily available due to technical limitations. Requests to access the datasets should be directed to the National Health Research Institutes.
